# A 12-Week-Long Intake of Bilberry Extract (*Vaccinium myrtillus* L.) Improved Objective Findings of Ciliary Muscle Contraction of the Eye: A Randomized, Double-Blind, Placebo-Controlled, Parallel-Group Comparison Trial

**DOI:** 10.3390/nu12030600

**Published:** 2020-02-25

**Authors:** Marie Kosehira, Naomichi Machida, Nobuyoshi Kitaichi

**Affiliations:** 1 Research Department, Omnica Co., Ltd, 4-21-7 Itabashi, Itabashi Ward, Tokyo 173-0004, Japan; 2 Department of Ophthalmology, Health Sciences University of Hokkaido, Sapporo 002-8072, Japan; nobukita@hoku-iryo-u.ac.jp

**Keywords:** bilberry, asthenopia, clinical trial, accommodation, refraction, accommodative microfluctuation

## Abstract

A 12-week-long randomized, double-blind, placebo-controlled, parallel-group comparison trial was conducted to determine the effects of long-term standardized bilberry extract (SBE) intake on tonic accommodation of ciliary muscle caused by visual display terminal (VDT) tasks. This study was compliant with the accordance with CONSORT 2010 statement. A total of 109 healthy adult men and women aged 20–60 years were recruited and randomized into SBE and placebo groups. The subjects in the SBE and placebo groups were administered 240 mg of SBE and placebo, respectively, once daily for 12 weeks. Tests were performed before and after VDT tasks at week 0, 4, 8, and 12; high-frequency component (HFC)-1 value was the evaluation outcome. Results showed that post-load HFC-1 values at weeks 8 and 12 were significantly improved in the SBE group than in the placebo group (*p* = 0.014 and 0.017, respectively). This study shows that oral consumption of 240 mg SBE extract for 12 weeks relieves the tonic accommodation of the ciliary muscle caused by VDT tasks and near-vision tasks.

## 1. Introduction

Owing to the increased distribution of visual display terminal (VDT) devices, our eyes are constantly being overworked [1,2]. According to the survey conducted by the Ministry of Health, Labour and Welfare, the proportion of workers performing VDT tasks is increasing; as per the 2008 survey, approximately 50% of all workers were found to be engaged in VDT tasks for ≥4 h/day. Of the workers experiencing physical fatigue and VDT work-related symptoms, 91.6% experience “ocular fatigue and ocular pain,” indicating a high load of VDT tasks on the eyes [1]. Recently, the use of VDT devices, such as smartphones and tablets when used and placed close to the eyes, is rapidly spreading among people. Individual smartphone ownership has quadrupled from 14.6% in 2011 to 56.8% in 2016, and ≥90% smartphone users are 40 years old or younger [3]. Because individuals use VDT devices, such as personal computers and smartphones, close to their eyes for long periods of time, the eye muscles become fatigued and experience tension, making focus adjustment difficult. Further, because more and more young people are starting to experience symptoms similar to presbyopia, it is extremely important to prevent and reduce ocular fatigue to improve the quality of life of VDT users.

Bilberry (*Vaccinium myrtillus* L.) contains 15 types of low-molecular anthocyanin glycosides made up of a combination of five types of aglycones (cyanidin, delphinidin, malvidin, peonidin, and petunidin) and three types of monosaccharides (glucose, galactose, and arabinose). These 15 *V. myrtillus* anthocyanins (VMAs) serve as the main components of bilberry. Bilberry extract obtained via the separation and refinement of these 15 VMAs has been used as a component of functional foods wherein the “tertiary” function of the food [4] is intentionally regulated instead of just consuming the bilberry. Different extraction methods yield bilberry extracts with different overall compositions; even if the bilberry extracts share the same VMAs, their benefits and safety could vary [5,6]. In the present paper, we refer to bilberry extract that has had reproducibility of effect confirmed in several tests [7,8,9,10] as standardized bilberry extract (SBE).

We confirmed that SBE consumption suppresses or relieves convergence, miosis, and declined accommodation function caused by VDT tasks [7,8,9,10]. Furthermore, we established intervention conditions for the tonic accommodation of the ciliary muscle caused by VDT tasks and verified the minimum effective dose of SBE [8] and minimum period for efficacy expression [9]. This 12-week randomized, double-blind, placebo-controlled, parallel-group comparison trial examining SBE consumption aimed to determine the effects and safety of long-term SBE consumption on the tonic accommodation of ciliary muscles caused by VDT tasks. 

This is the first trial for the efficacy of Bilberry extract in accordance with the CONSORT 2010 statement (Figure S1, Table S1).

## 2. Methods and Subjects

### 2.1. Trial Design and Subjects

To evaluate the effect of SBE consumption on ocular fatigue symptoms, we conducted a randomized, double-blind, placebo-controlled, parallel group human clinical trial. The trial strictly adhered to tenets of the Declaration of Helsinki and the Ethical Guidelines for Medical and Health Research Involving Human Subjects, and the trial plan was approved by the Huma R&D ethical review board (approval date 15 January 2016). This study was registered at https://www.umin.ac.jp/ctr/index.htm (UMIN registration number: UMIN000020866). All subjects were provided with a sufficient explanation before participation in the trial, and written informed consent was obtained. The ocular fatigue test was performed by the Medicine Evaluation Research Center, whereas the safety test was performed by the K Medical Office TOC Building Clinic.

A total of 109 healthy adult men and women aged 20–60 years responded to the Human R&D public request for trial participation. Subjects whose jobs involved VDT tasks and those who had subjective ocular fatigue symptoms were selected. Interviews, body measurements, blood tests, urine tests, and ocular fatigue tests were conducted during the pretrial screening, and preliminary questionnaires were administered. Subjects who met any of the following criteria were excluded from the trial:

1. Serious diseases of the digestive system, liver, pancreas, heart, or kidney, or a history of such a disease
2. currently under treatment with pharmaceuticals
3. ocular diseases other than ametropia (hyperopia, myopia, or astigmatism)
4. existing drug and food allergies
5. history of, or under treatment for, drug dependence and substance abuse
6. nightshift or irregular work hours
7. history of refractive surgeries; LASIK (laser-assisted in situ keratomileusis) or others
8. users of reading glasses
9. current consumption of BE, pharmaceuticals, or supplements that may improve ocular fatigue
10. participated in another clinical trial within the last 1 month
11. pregnant, breastfeeding, or planning for pregnancy during the trial period
12. blood sampling of ≥ 200 mL within the last 1 month, and
13. deemed illegible by the trial supervisor or trial attending physician

During the pretrial screening, the subjects’ dominant eyes were determined using the hole-in-the-card method. The subjects were randomized into SBE and placebo groups by staff who were not involved in the intervention trial; sex and age were balanced between the groups. The staff blinded the subjects by masking the identification of tested foods with symbols, and the tested foods were securely managed by the same staff.

According to our previous trials on SBE consumption [8], high-frequency component (HFC) of accommodative microfluctuation was measured. We calculated the power spectrum of HFC based on measured values using fast Fourier transformation [11]. The significance level was set at <5%. Under this condition, the number of subjects required to set the statistical power at 0.8 or 0.9 was 88 or 117, respectively. The goal case number was chosen as 120 such that the statistical power would be ≥0.8, even if the subjects dropped out during the trial period.

### 2.2. Test Foods

The SBE group was administered a hard capsule containing 240 mg SBE (Omnica Co., Ltd, Tokyo, Japan), which was the same SBE as used in previous reports [7,8,9,10]. The placebo group was administered a control capsule without SBE. Table 1 shows the composition of the tested foods. The concentration of VMA in SBE was 36.89% when determined using HPLC with cyanidin 3-glucoside chloride as the anthocyanin reference and 35.82% when determined using a quantitative method that employs the absolute purity of the reference material. The true value of cyanidin 3-glucoside chloride in SBE measured by converting the reference material of absolute purity was 3.83% and the true concentration of a total of 15 VMAs was 29.00%. Approximately 68.0 mg of caramel color was added to the placebo food to differentiate it from the SBE food.

### 2.3. Consumption Methods and Schedule

The subjects consumed SBE or placebo food once daily for 12 weeks without changing their daily routine. The SBE and placebo foods were to be consumed on an empty stomach before breakfast or lunch.

**Figure 1:**
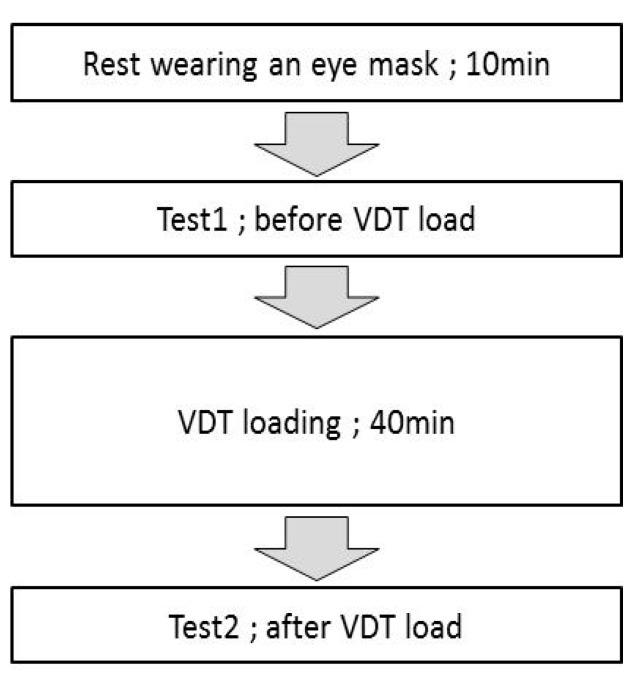
Test schedule.

Figure 1 shows the flow of tests performed before and after the VDT tasks at week 0, 4, 8, and 12. Each subject wore an eye mask upon arrival at the trial venue, rested for 10 min, and then had their HFC of accommodative microfluctuation measured before VDT loading. Measurements were made in the order of the dominant eye followed by the nondominant eye.

Before the load test, the subjects performed VDT tasks for 40 min in a room with an illuminance of 500 ± 50 lux. The load task was as follows. A string was attached to a smartphone (Apple Inc. iPhone 5), which was looped over the subjects’ necks such that the screen and the subjects’ eyes were 30 cm apart. The subjects played a game in this position such that they all had a similar VDT load. The game was a simple one of collecting falling debris: Tetris®. As the post-load test, HFC was measured 1 min after the end of the VDT task. 

### 2.4. Test Items

The ocular tests (HFC measurements) were performed at week 0-the first day of intervention-(0w), week 4 (4w), week 8 (8w), and week 12 (12w). The clinical diary was checked daily (consumption of the tested food, health condition, adverse events, etc.). The safety test was performed during the screening (SCR) and at week 12 (Table 2).

#### 2.4.1. Ocular Fatigue Test

Using the ocular accommodation function analysis software AA-2 (NIDEK, Nagoya, Japan) installed in the Autorefractor/keratometer ARK-560A (NIDEK), the HFC was examined according to the report by Kajita et al. [12]. In brief, the accommodation response waveform was measured 8 times by changing the position of the target at the 0.5 diopter interval from far to near positions between +0.5 and −3.0D based on the minimum refraction calculated for each measurement. Examinees were instructed to maintain a clear gaze at the target at each step. Frequency spectra, obtained through fast Fourier transform of the obtained waveforms, were converted to logarithm, and this was integrated by the frequency of 1.0–2.3 Hz to obtain HFC. The fraction from the minimum refraction to −0.75D was defined as the HFC-1 range, and the mean HFC in the HFC-1 range was defined as the HFC-1 value. When human eyes are fixed upon a stationary target, subjectively, refraction seems to be stationary; however, in reality, the ciliary muscle repeatedly contracts and relaxes, leading to a sinusoidal fluctuation in refraction. This fluctuation is called accommodative microfluctuation and is divided into low frequency components (gradual fluctuations of <0.6 Hz) and high-frequency components (relatively fast fluctuations of 1.0–2.3 Hz). The low frequency components are generated by accommodation that adjusts the point of focus, whereas the high-frequency components originate from fluctuations in the refractive power of the lens, reflecting the activity of the ciliary muscle [11,13,14].

#### 2.4.2. Safety Evaluation

For safety evaluation, blood tests, urine tests, body measurements, and interviews were conducted before and after the start of the trial. All undesirable or unintentional signs, symptoms, and illnesses that occurred during the consumption of the tested foods in this trial were considered as adverse events, regardless of the causal relationship with the tested food.

### 2.5. Statistical Processing

Analysis of covariance (ANCOVA) was used to compare the HFC-1 values between the groups. Values < 10% were considered a significant trend, and those < 5% were considered a significantdifference in two-tailed tests.

Data were analyzed using SAS 9.3 (SAS Institute, Inc, Cary, NC, USA) and Microsoft Excel 2013 (Microsoft Corp.).

## 3. Results

### 3.1. Subject Background

The subjects were recruited between January 18 and February 29, 2016, and the trial was conducted from April 11 to July 21, 2016. Of the 206 subjects who underwent pretrial primary screening using blood and urine tests as well as a questionnaire, 175 were selected to proceed to the secondary screening. Of these 175 subjects, 14 people were declined from the study for personal reasons and 161 underwent HFC-1 measurement as the secondary screening; from these 161 subjects, 120 were determined to be eligible based on their results.

**Figure 2:**
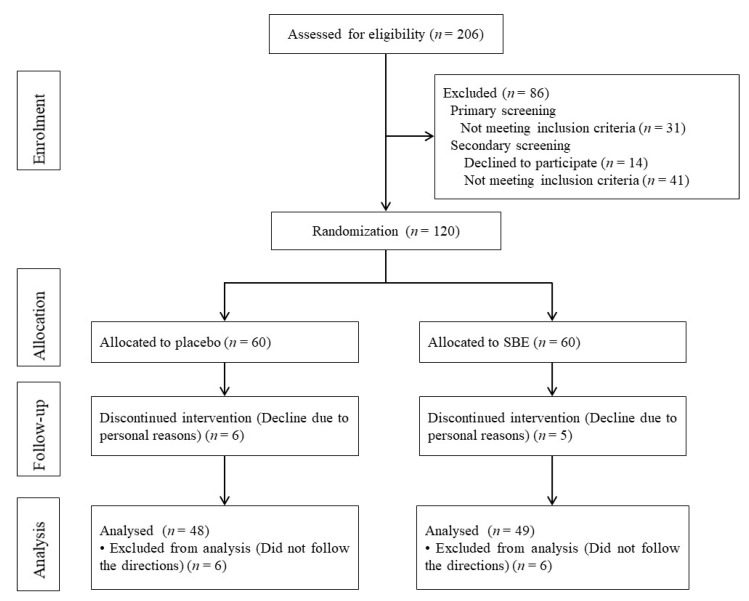
Flow chart of the study (*n* = total subjects).

Of the 120 eligible subjects, 38 were men and 82 were women; the mean age of the subjects was 35.16 ± 7.38 years. Before the trial started, 11 subjects withdrew due to personal reasons and the remaining 109 subjects (55 in the SBE group and 54 in the placebo group) completed the prescribed schedule and trial. Table 3 presents the background information of the 109 subjects. For the HFC-1 analysis, 97 out of 109 were able to complete the trial according to the instructions provided by the tester. The age distribution of the subjects was 10 subjects in their 20s, 18 subjects in their 30s, and 20 subjects in their 40s in the placebo group and 10 subjects in their 20s, 14 subjects in their 30s, and 25 subjects in their 40s in the SBE group. Therefore, the age distribution of both groups is similar and there was no difference in lifestyle. Figure 2 presents the flow from enrollment of the subjects to analysis of the data. 

### 3.2. Impact on Ocular Fatigue Based on HFC-1 Values

**Figure 3:**
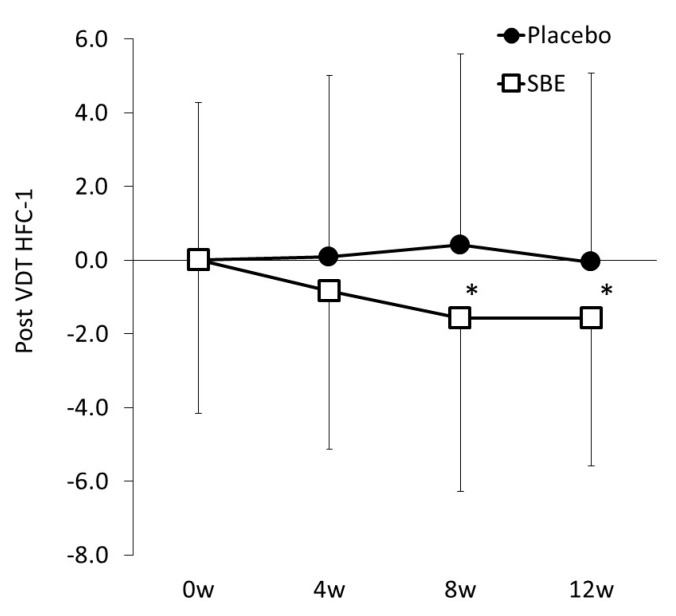
Value changes at week 0, 4, 8, and 12. Changes in the HFC-1 values. Statistical significance: * *p* < 0.05 versus placebo group.

As for the difference between the before-load and post-load HFC-1 values (ΔHFC-1), when the variation at week 0 (SBE versus placebo, 1.135 versus 0.123) was covariant, the SBE group presented significantly lower values than the placebo group (Table 4 and Figure 3B) at week 4 (SBE versus placebo, 0.478 versus 1.296, *p* = 0.018) and week 12 (SBE versus placebo, 0.053 versus 0.347, *p* = 0.049).

### 3.3. Safety

The incidences of adverse events during the trial period were surveyed based on the subjects’ (*n* = 109) journals and interviews by physicians. Multiple occurrences of the same adverse event in the same subject were considered as one case (Table 5). All adverse events that occurred were determined to be unrelated to the tested foods by the physicians. In addition, the physicians determined that there were no changes in the general blood, blood biochemistry, and urine tests associated with the tested food consumption. 

## 4. Discussion

In the present study, suppression of and relief in ocular fatigue, with ciliary muscle contraction caused by VDT load as the index, was confirmed. This is one of the large-scale randomized, double-blind, placebo-controlled clinical trials concerning ocular fatigue that have been conducted in recent years. The subjects that were included in the present trial were healthy individuals with ocular fatigue. The subjects in the SBE group consumed 240 mg of SBE once daily for 12 weeks. 

With the minimum refraction of each subject as a reference, we moved the target from the far to the near positions in eight steps and measured the frequency of HFC of accommodative microfluctuation. These values were digitized by the “Accommodative function measurement software AA-2” as HFC [15]. HFC is generated via vibration of the ciliary muscle and increases with excessive load [16,17]. In a trial that examined the subjective symptoms via a simple questionnaire and measured HFC values, compared with subjects who did not experience much ocular fatigue, those who routinely experienced ocular fatigue had a greater increase in HFC following VDT tasks. In addition, the subjects who responded that VDT tasks either fatigued or extremely fatigued their eyes demonstrated a greater increase in HFC compared with subjects who only experienced mild or little ocular fatigue [18]. As such, HFC is sufficiently and specifically correlated with subjective ocular fatigue and is widely used to evaluate ocular fatigue [12,19,20]. In addition, we used HFC to confirm the effects of SBE consumption on ocular fatigue [8,9]. In the present trial, we defined the range from the minimum refraction to −0.75 D as the HFC-1 range and used the mean HFC in the HFC-1 range fraction to evaluate ocular fatigue. To eliminate the impact of pre-consumption values in each group, we performed intergroup comparison using ANCOVA.

Due to the continuous consumption of SBE, compared with the placebo group, the post-VDT load HFC-1 values showed a significant improvement at weeks 8 and 12, suggesting that SBE suppresses the tonic accommodation of ciliary muscles in response to VDT load. The observable effects from weeks 8 to 12 of SBE consumption indicate that 12 week long-term consumption of SBE would not attenuate its efficacy.

Anthocyanin in bilberry extract is a highly soluble antioxidant with a flavylium ion structure [21]. It is found in the ocular tissues [22] and suppresses various inflammatory factors in the body [23]. Because it is possible that anthocyanins migrate and are distributed from the blood to the ciliary muscle, they can suppress and relieve the tension in the ciliary muscle, thereby potentially reducing ocular fatigue.

In conclusion, the present trial demonstrated that 12-week oral consumption of 240 mg of SBE ameliorates and relieves the tonic accommodation of ciliary muscles caused by VDT tasks and near-vision tasks.

## Figures and Tables

**Table 1:** Composition of standardized bilberry extract (SBE) and placebo foods (mg/day).

	SBE Food	Placebo Food
SBE *	240.0	0
Dextrin	27.2	51.0
Starch	45.6	193.8
Calcium stearate	22.1	22.1
Silicon dioxide (fine)	5.1	5.1
Caramel color	0	68

* Functional components: total anthocyanins of 36.89%.

**Table 2:** Tested foods and test schedule.

Examination period	Screening	0 week	4 weeks	8 weeks	12 weeks
Ophthalmic examination (HFC)	●	●	●	●	●
Anthropometric	●				●
Doctor’s questions	●				●
Blood test/Urinary test	●				●
Diary		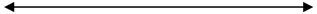			

Test points: ●, Diary entry period: 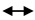.

**Table 3:** Subjects’ background information.

Variables	Placebo	SBE
Gender (male/female) (*n*)	54 (16/38)	55 (18/37)
Age (years)	35.46 ± 6.96	36.18 ± 7.14
Height (cm)	163.72 ± 8.03	163.11 ± 8.71
Body weight (kg)	56.78 ± 8.71	57.77 ± 10.65
Body mass index (kg/m^2)^	21.06 ± 2.3	21.58 ± 3.13

**Table 4:** HFC-1 values.

		Placebo (*n* = 48)			SBE (*n* = 49)			*p* Value
		Mean	±	SD	Mean	±	SD	
0w	Before VDT load	50.60	±	4.13	51.08	±	3.84	
	Post VDT load	50.72	±	4.28	52.21	±	4.16	
4w	Before VDT load	49.51	±	4.13	50.90	±	5.04	
	Post VDT load	50.81	±	4.92	51.37	±	4.29	0.225
8w	Before VDT load	50.94	±	4.93	50.08	±	4.68	
	Post VDT load	51.13	±	5.19	50.64	±	4.70	* 0.014
12w	Before VDT load	50.32	±	4.33	50.59	±	4.20	
	Post VDT load	50.67	±	5.13	50.65	±	4.03	* 0.017

Values are shown as the means ± standard deviations; Before VDT load HFC-1 and Post VDT load HFC-1. Statistical significance: * *p* < 0.05 versus placebo group. VDT: visual display terminal.

**Table 5:** Adverse events in the standardized bilberry extract (SBE) and placebo groups.

Adverse Events	Placebo (*n* = 54)	SBE (*n* = 55)
Common cold	12	10
Headaches	11	18
Digestive symptoms (stomachache or diarrhea)	6	6
Menstrual pain	3	4
Other	17	24

## Supplementary Materials

The following are available online at https://www.mdpi.com/2072-6643/12/3/600/s1, Figure S1: CONSORT 2010 Flow Diagram, Table S1: CONSORT 2010 checklist of information to include when reporting a randomised trial.

## Abbreviations

HFC	high-frequency component
SBE	standardized bilberry extract
VDT	visual display terminals
VMA	*Vaccinium myrtillus* anthocyanin
